# Upregulation of Somatostatin Receptor Type 2 Improves ^177^Lu-DOTATATE Therapy in Receptor-Deficient Pancreatic Neuroendocrine Tumor Model

**DOI:** 10.1158/1535-7163.MCT-22-0798

**Published:** 2023-07-24

**Authors:** Rupali Sharma, Bhargav Earla, Kwamena E. Baidoo, Martha A. Zeiger, James P. Madigan, Freddy E. Escorcia, Samira M. Sadowski

**Affiliations:** 1 Endocrine Surgery Section, Surgical Oncology Program, Center for Cancer Research, NCI, NIH, Bethesda, Maryland.; 2 UAB Heersink School of Medicine, Birmingham, Alabama.; 3 Molecular Imaging Branch, Radiation Oncology Branch, Center for Cancer Research, NCI, NIH, Bethesda, Maryland.; 4 Office of Surgeon Scientists Programs, Center for Cancer Research, NCI, NIH, Bethesda, Maryland.

## Abstract

Pancreatic neuroendocrine tumors (PNET) express high levels of somatostatin receptor type 2 (SSTR2), a unique target for both tumor imaging and therapy. This surface expression is lost in metastatic high-grade PNETs, making patients ineligible for SSTR2-targeted ^177^ Lutetium (Lu)-DOTATATE peptide receptor radionuclide therapy (PRRT), and represents an unmet clinical need. Here, we aimed to restore SSTR2 expression through the reversal of inhibitory epigenetic gene silencing to improve tumor responsiveness to PRRT. We first assessed human SSTR2 promoter methylation and expression levels in 96 patient samples. We then used three NET cell lines (QGP-1, BON-1, GOT-1) with variable SSTR2 expression profiles for functional *in vitro* studies using histone deacetylase inhibitors (HDACi). Finally, the QGP-1 xenograft mouse model, with low basal SSTR2 expression, was used to assess the therapeutic efficacy of combined HDACi and ^177^Lu-DOTATATE therapies. We confirm that SSTR expression is decreased and correlates with SSTR2 promoter methylation in patients with high-grade NETs. When exposed to HDACis, SSTR2 surface expression is increased in three NET cell lines *in vitro*. In an *in vivo* PNET xenograft model with low basal SSTR2 expression, our studies demonstrate significantly higher tumor uptake of SSTR2-targeted ^177^Lu-DOTATATE in animals pretreated with HDACis compared with controls. For the first time, we show that this higher tumor uptake results in significant antitumor response when compared with standard PRRT alone. These preclinical results provide a rationale for utilizing HDACi pretreatment to improve targeted radionuclide therapy in patients with SSTR2-negative, metastatic PNETs.

## Introduction

While gastro-entero-pancreatic neuroendocrine tumors (GEP-NET) are rare, their incidence in the United States has increased 6.4-fold over the last four decades (1). Overall survival (OS) in high-grade, metastatic NETs is less than 25% (2, 3, 4). High-grade, poorly differentiated tumors are associated with poor prognoses (5) and are characterized by a loss of somatostatin receptor type 2 (SSTR2) expression (6). These SSTR2-negative tumors are refractory to targeted therapies, respond poorly to chemotherapy (streptozocin- or platinum-based; ref. 7), and patients with these aggressive tumors have no effective treatments available.

Our prior work contributed to the approval of the PET-imaging agent, ^68^Ga-DOTATATE (6, 8). This agent is a high-affinity ligand that binds SSTR2, and has not only revolutionized the diagnosis of low-grade NETs, but also allows for peptide receptor radionuclide therapy (PRRT) with ^177^ Lutetium (Lu)-DOTATATE (9). However, PRRT is ineffective in patients with high-grade NETs because these tumors exhibit low or no levels of SSTR2 expression. We posit that pharmacologically restoring or increasing SSTR2 expression of receptor-negative NETs could render these tumors responsive to PRRT.

Epigenetic regulation is one of several mechanisms used by cells to modulate gene expression. DNA methylation and specific histone modifications can lead to transcriptional inactivation of tumor suppressor genes and are directly correlated with tumorigenesis of GEP-NETs (10). No genomic alterations have been found in the *SSTR2* gene of NETs. In addition, NETs have a low mutation burden, and increased DNA methylation correlates with poor patient outcomes. Thus, epigenetic regulation may play a dominant role in the oncogenesis and progression of NETs, and account for the lack of SSTR2 in high-grade NETs (11). Several preclinical studies have shown that epidrugs, so-called epigenetic enzyme inhibitors, significantly increased SSTR2 expression in NET cell lines with low basal expression (12, 13, 14). However, translation of this phenomenon to *in vivo* animal models using combination therapies has not been previously demonstrated.

In this study, we first confirm increase in SSTR2 expression in three GEP-NET cell lines (QGP-1, BON-1, and GOT-1) using targeted epidrug treatment [the histone deacetylase inhibitors (HDACi), valproic acid (VPA), and tacedinaline (CI-994), and the DNA methyltransferase inhibitors (DNMTi), azacitidine, and decitabine]. Furthermore, we establish the translational potential of these findings by demonstrating that the HDACi CI-994 can both increase *in vivo* uptake of ^177^Lu-DOTATATE and, for the first time, confirm the concomitant therapeutic efficacy of PRRT with ^177^Lu-DOTATATE in a preclinical model of SSTR2-deficient pancreatic NETs (PNET).

## Materials and Methods

### Compounds

HDACis, VPA and CI-994, and DNMTi, decitabine, were purchased from Sigma-Aldrich and Azacitidine from Selleckchem. CI-994 [acetyldinaline; 4-acetylamino-N-(2′aminophenyl)-benzamide] is the acetylated metabolite of dinaline and its chemical structure can be found in reference (15). CI-994 and decitabine were dissolved in DMSO (Sigma-Aldrich), VPA dissolved in water and, all stored at –20°C. Concentrations of each inhibitor were calculated below the MTD used in human trials (16, 17, 18, 19). ^177^LuCl_3_ was obtained from MURR (MU Research Reactor, University of Missouri, Columbia, MO). ^177^Lu-DOTATATE was synthesized as previously described (20).

### EPIC/850k methylation array and RNA sequencing in human samples and cell lines

GEP-NETs samples were collected from patients under a tissue procurement protocol at the NIH Clinical Center after Institutional Review Board (IRB) approval of the protocol by the Center for Cancer Research Center, NCI (IRB 09-C-0242). The samples were scored using the World Health Organization (WHO) classification system (21). 850K/EPIC array data in a cohort of 96 samples of NETs from NIH were analyzed using R-based platforms (22). Methylation levels among different tumor grades were analyzed using the two-tailed *t* test. The Chan and colleagues dataset (23), GSE117852, containing 32 human PNET samples, was analyzed using Spearman method, with the *P*-value based on the rank of the expression. 850K/EPIC array analysis and RNA sequencing (RNA-seq) were performed in QGP-1, BON-1, and GOT-1 cells after a 4-day treatment with VPA and azacytidine. Normalized methylation data were evaluated in R using Champ package functions (champ.norm).

### Cell culture

Three NET cell lines were used: QGP-1, BON-1, and GOT-1. BON-1 was established from a lymph node metastasis from a PNET patient (University of Texas Medical Branch at Galveston, Galveston, TX; ref. 24). QGP-1 was established from a somatostatin-producing pancreatic islet cell carcinoma (25), purchased from the Japanese Collection of Research Bioresources cell bank. The GOT-1 cell line was established from a liver metastasis of a primary intestinal NET (Sahlgrenska Cancer Center, University of Gothenburg, Gothenburg, Sweden; refs. 26, 27). Cell lines were authenticated by STR analysis via LabCorp's human cell line authentication service and routinely tested for *Mycoplasma* infection using the MycoAlert PLUS Mycoplasma Detection Kit (LT07–705, Lonza).

### RNA extraction and real-time RT-PCR

For mRNA analysis, total RNA was isolated using RNeasy Plus Mini kit (#74106, Qiagen). The RNA concentrations were determined using a NanoDrop 1000 spectrophotometer (Thermo Fisher Scientific) and 600 ng of RNA were used to synthesize cDNA using a High-Capacity cDNA Reverse Transcription Kit (Thermo Fisher Scientific). The sequences of the PCR primers used in this experiment are as follows: SSTR2 Hs00265624_s1 and β-actin Hs01060665_g1. qRT-PCR was performed in triplicate on a Thermo Fisher Scientific Quant Studio 5 Real-Time PCR System (Thermo Fisher Scientific). Target gene expression was normalized to β-actin, and the ΔΔ*C*_t_ method was used to calculate relative gene expression.

### Cell surface expression of SSTR2

To analyze cell membrane expression of SSTR2, QGP-1, BON-1, and GOT-1 cell lines were treated for up to 5 days with HDACi. After treatment, the cells were fixed in 4% paraformaldehyde (PFA) and resuspended in flow buffer (0.5% BSA in sterile PBS). Then, 1 × 10^6^ cells per replicate were stained in a dark room for 30 minutes at room temperature with an anti-SSTR2 (IC4224G, 1 μg/5 μL) antibody (R&D Systems; Supplementary Table S1). Cells were then analyzed by flow cytometry using a BD LSR-Fortessa analyzer (BD Biosciences) and data analyzed using FlowJo V10.6.2 (Tree Star, Inc.).

### Western blot analysis

Cells were treated, lysed with GPCR Extraction and Stabilization Reagent buffer (#A43436, Thermo Fisher Scientific), rotated at 4°C, and sonicated twice. Protein concentrations were determined using a Micro BCA Protein Assay Kit (#23235, Thermo Fisher Scientific). Fifty micrograms of protein lysate were resolved on Bolt 4%–12% Bis-Tris Plus Gels (#NW04120BOX, Life Technologies) and electrophoresed at 200V for 30 minutes. Gels were transferred to polyvinylidene difluoride membranes using the iBlot2 dry transfer method (# IB21001, Life Technologies). Membranes were blocked in 3% BSA/5% milk in 1× Tris buffer saline/0.05% Tween 20 (1× TBST) for 1 hour at room temperature followed by primary antibody incubation overnight, at 4°C, washed in 1× TBST, then incubated with a secondary antibody for 1 hour at room temperature. Membranes were then incubated with a chemiluminescent substrate (#34577, SuperSignal West Pico Chemiluminescent Substrate, Thermo Fisher Scientific) and imaged on the Bio-Rad Chemidoc Imaging System (Hercules). Densitometry was performed with NIH ImageJ software (2.0.0; ref. 28), normalized to GAPDH or β-actin.

#### SSTR2-specific antibody validation through SSTR2 stable knockdown

To stably knock down expression of human SSTR2, two separate pLKO.1-puro lentivirus plasmid–based shRNAs targeting the sequences TGAAGACCATCACCAACATTT (#64) and CCCTTCTACATATTCAACGTT (#87; human MISSION shRNA clone ID: TRCN0000358164, SSTR2 #64-shRNA, and human MISSION shRNA clone ID: TRCN0000014387, SSTR2 #87-shRNA, Sigma-Aldrich) were employed. In addition, a nontargeting shRNA plasmid (NT-shRNA) that targets no known human sequence was used as a control. A primer containing the target sequence (CTGGTTACGAAGCGAATCCTT), along with a stem-loop followed by the reverse target sequence, was annealed to a complementary primer and inserted into the *Eco*RI and *Age*I sites of pLKO.1-puro (#8453, Addgene). Lentiviral particles were produced via lipofectamine 2000 (Invitrogen)-mediated triple transfection of 293T cells with the respective pLKO.1-puro shRNA plasmids, along with the lentiviral envelope plasmid (pMD2.G, #12259, Addgene) and the lentiviral packaging plasmid (psPAX2, #12260 Addgene). BON-1 target cells were transduced with shRNA-containing lentiviral particles in the presence of 8 μg/mL Polybrene (Invitrogen), and stable cells were selected using 2 μg/mL puromycin (Invitrogen). Efficiency of *SSTR2* mRNA knockdown was determined using qRT-PCR. Western blot analysis of control and SSTR2-specific, stable knockdown BON-1 cell lysates was used to validate the efficacy and utility of a commercially available, SSTR2-specific antibody, M01689 (Boster Bio; Supplementary Table S1; Supplementary Fig. S1A and S1B).

### Mouse model

The animal protocol for this study was approved by the NCI/CCR Animal Care and Use Committee (ASP SOP-001). QGP-1 or BON-1 cells (5 × 10^6^ cells) were injected subcutaneously into both flanks of five- to seven-week-old athymic nude male mice CAnN.Cg-Foxn1nu/Cr, (The Jackson Laboratory). The resulting tumors were further subcutaneously implanted, to create homogenous tumors. Only tumors from generations 1 to 3 were utilized. Tumor size was monitored using a Vernier caliper, with tumor initiation size ranging from 5 to 7 mm. The mice were euthanized if they exhibited impeded movement, if there were signs of breathing difficulty or tumors exceeded 2 cm in diameter at any point in the study. Mice were randomly grouped into control and treatment groups and then treated via intraperitoneal daily injections with either VPA (300 mg/kg; P4543; Sigma-Aldrich) dissolved in water, CI-994 (5 mg, 7.5 mg, and 10 mg/kg; C0621; Sigma-Aldrich) dissolved in 0.1% DMSO, or 0.1% DMSO as control group. All interventions were done during the light cycle, and mice were not fasted before assessments.

### Cell surface expression of SSTR2

#### Flow cytometry

For flow cytometry analysis, mice were treated with DMSO, VPA (300 mg/kg), or CI-994 (10 mg/kg). At the end of the treatment period, blood was collected by cardiac puncture and the tumors were harvested for FACS analysis. The xenograft tumors were dissociated using a human tumor dissociation kit (#130–095–929, Miltenyi Biotec), 1 × 10^6^ cells were stained with an Alexa Fluor 488–conjugated anti-SSTR2 (1 μg/5 μL) antibody (IC4224G, R&D Systems) and were analyzed for surface expression of SSTR2 using a BD LSR-Fortessa analyzer (BD Biosciences). Alexa Fluor 488–conjugated mouse IgG2A antibody (1 μg/5 μL; IC003G, R&D Systems) was used as an isotype control. Data were analyzed using FlowJo V10.6.2 (Tree Star, Inc.).

#### Receptor saturation assay

Receptor assay was performed as per Panke and colleagues (29). Briefly, Molecules of Equivalent Soluble Fluorophore (MESF) calibration beads for Alexa Fluor 488 were purchased - Quantum TM MESF beads (488A, Bangs laboratories). MESF beads median values of fluorescence were calculated on BD LSR-Fortessa analyzer (BD Biosciences). Using the same settings, Fluorescence/Protein (F/P) ratio for SSTR2 antibody was calculated using Simply Cellular (810, Bangs laboratories). BON1 and QGP1 were treated with DMSO and CI-994, stained with Alexa Fluor 488-conjugated anti-SSTR2 (1 μg/5 μL) antibody (IC4224G, R&D Systems) and were run on same settings. Median MFI of SSTR2 staining on SSTR2 expressing cells was calculated and converted for the number of antibody bound per cell, displayed as median number of antibody/cell. Data were analyzed using FlowJo V10.6.2 (Tree Star, Inc.).

#### Immunofluorescence

Immunofluorescence (IF) staining was performed on commercially available pancreas tissue slides (Islet Cell Tumor NBP2–77922, Novus Biologicals), as a positive control for SSTR2, and on formalin-fixed, paraffin-embedded (FFPE) mouse tumor samples. Slides were incubated with the primary antibody for SSTR2 (UMB1; ab134152; 1:25; Abcam) and a secondary antibody, Alexa Fluor 594 (A11037;1:500, Invitrogen); For Pan-acetylated H3 primary antibody (06–599; 1:500; EMD Millipore) and secondary antibody (AF594; 1:100, Thermo Fisher Scientific); For γH2AX primary antibody (05–636–1; 1:800; EMD Millipore) and secondary antibody (AF488; 1:100, Thermo Fisher Scientific; Supplementary Table S1). Zeiss LSM - 8800 Confocal microscope was used for images.

#### γH2Ax counting

4',6-diamidino-2-phenylindole (DAPI)-stained nuclei were segmented using the Cellpose DL package (30). A custom Cellpose model using Biowulf HPC computational resources (http://hpc.nih.gov) was used for nuclei segmentation. These nuclei segments were then used for γH2Ax intensity quantification.

### Biodistribution studies

Mice were euthanized 24 hours post-^177^Lutetium (Lu)-DOTATATE injection (2 MBq/mouse). In addition to the tumors, the following organs were harvested: heart, lungs, liver, spleen, kidneys, stomach, small and large intestine, muscle, and femur. The counts per minute (CPM) readings, obtained using a 2480 automatic gamma counter (Perkin Elmer), were normalized by tumor weight and biodistribution data were presented as the percentage of the injected dose per gram of tissue (%ID/g).

### Peptide receptor radionuclide therapy

To evaluate the therapeutic efficacy of the treatments, the radionuclide ^177^Lu was labelled with DOTATATE (#H-6318005; Bachem). Both BON-1 and QGP-1 tumor mouse models were pretreated with either CI-994 or DMSO for 10 days. The mice were then randomized into the following treatment groups: (i) saline; (ii) saline followed by ^177^Lu-DOTATATE (30 MBq/mouse); (iii) CI-994 followed by saline; and (iv) CI-994 followed by ^177^Lu-DOTATATE (30 MBq/mouse). Five minutes prior to ^177^Lu-DOTATATE administration, D-lysine hydrochloride (35 mg/mouse; #243080010, Thermo Fisher Scientific) was given to block kidney uptake and prevent nephrotoxicity from ^177^Lu (31). The tumor burden in each mouse was monitored twice a week and calculated using the formula tumor volume = (length × width^2^)/2.

### Statistical analysis

GraphPad Prism 8.1 (GraphPad Software, Inc.) software was used for data analysis. Two-way ANOVA was used to analyze qPCR and flow cytometry data to determine SSTR2 expression differences between the treatment groups. A two-tailed, unpaired Student *t* test was used for comparisons between two groups. One-way ANOVA with a *post hoc* Tukey test was used for comparisons between more than two groups. Two-way ANOVA with interaction effects was performed at different timepoints for the therapeutic study *P* value was calculated using two-tailed Mann–Whitney *U* test in R software to compare the DNA damage between the two groups. Data are presented as mean ± SEM, error bars show SEM. A *P* < 0.05 was considered significant.

### Data availability

Datasets generated and/or analyzed during the current study are available from the corresponding author on reasonable request. Array data of patients with NETs from NIH are deposited in the Gene Expression Omnibus (GEO; ref. 22; GSE134089).

### Study approval

All procedures were carried out in accordance with the NIH IC Animal Care & Use Committee guidelines, as well as Institutional radiation guidelines, and were approved by the NCI/CCR Animal Care and Use Committee (ASP SOP-001) and the NIH Radiation Safety Committee, Bethesda, MD.

## Results

### SSTR2 promoter methylation varies according to tumor grade in GEP-NET patients

**Figure 1.**
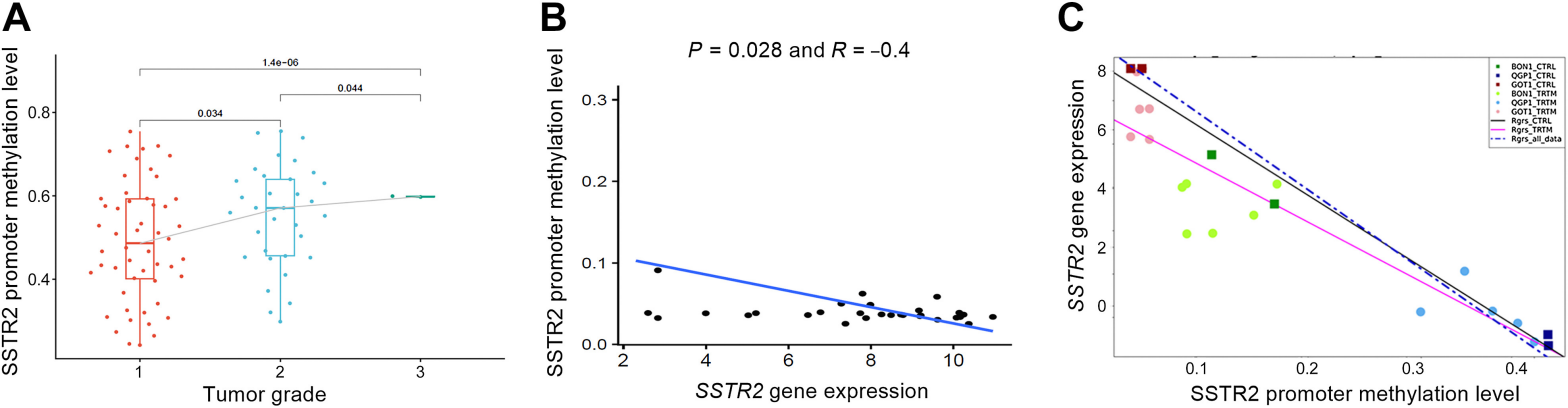
*SSTR2* promoter methylation levels in GEP-NET patients (**A** and **B**) and three NET cell lines (**C**). **A,** An NIH cohort of 96 patients with GEP-NETs was analyzed on the basis of tumor grade and methylation level. Those patients with higher-grade tumors showed increased methylation levels at the CpG methylation sites of the SSTR2 promoter. Significant changes were observed in the methylation levels between grades 1 and 2 (*P* = 0.034), grades 2 and 3 (*P* = 0.044), and grades 1 and 3 (*P* = 0.000014). **B,** The GSE117852 dataset of 32 patients with PNETs was analyzed for gene expression and mean *SSTR2* promoter methylation levels, using the Spearman method to calculate the *R* and *P* value. **C,** Methylation levels and SSTR2 expression were measured before and after treatment with VPA and azacytidine (TRTM) in three NET cell lines (QGP-1, BON-1, GOT-1). The controls are untreated for comparison (CTRL). The *x*-axis represents the degree of methylation (0–1), adjusted graphically for optimal visual display. The most significant changes were observed at CpG island cg14232289 (*P* < 0.0001).

Correlating methylation levels of 12 CpG methylation sites at the *SSTR2* promoter region with tumor grades in 96 NIH patient samples, we found increased methylation levels at higher tumor grades, as shown for one of the CpG methylation sites (cg19129425; *P* < 0.05) in Fig. 1A. This indicates closed chromatin with suppressed gene expression in high-grade tumors. The International Cancer Genome Consortium (ICGC; 450K EPIC array) and NIH data (850K EPIC array) sets were merged to examine tumor grades and methylation levels. Significant differences were observed in the methylation levels between grade 1 and 2 tumors (*P* = 0.034), but not with grade 3 tumors. Perhaps this is due to the small sample size for grade 3 tumors (very rare, *n* = 2), which makes it difficult to obtain statistically relevant information. Analysis of the GSE 117852 dataset of 32 PNET samples showed a significant negative correlation between *SSTR2* gene expression and mean *SSTR2* promoter methylation levels (Fig. 1B), indicating low mean promoter methylation levels (open chromatin) in tumor samples exhibiting elevated SSTR2 expression.

### SSTR2 promoter methylation correlates with SSTR2 expression and segregates according to GEP-NET cell line type

When correlating the methylation levels of 27 CpG methylation sites at the *SSTR2* promoter regions with SSTR2 expression in three NET cell lines, we found variable SSTR2 expression and a significant negative correlation between the levels of SSTR2 expression and DNA methylation at 11 CpG sites (cg14232289: *P* < 0.0001; Fig. 1C). Significant differences were observed across all three cell lines in their baseline SSTR2 methylation/expression levels and segregated according to cell line types (Fig. 1C, baseline expression = rectangular dots). VPA- and azacytidine-induced methylation changes were seen in the QGP-1 cells, with concomitant changes in SSTR2 expression (Fig. 1C, blue circles). Overall, QGP-1 cells showed the lowest baseline expression of SSTR2 when compared with BON-1 and GOT-1 cells, with correspondingly higher *SSTR2* promoter CpG methylation.

### HDACis VPA and CI-994 upregulate SSTR2 expression

Our inhibitor studies (Fig. 1C) demonstrated that treatment with an HDACi decreased *SSTR2* promoter CpG methylation levels. An important feature of epigenetic regulation is the interconnectedness of disparate epigenetic features and their enzymatic proteins (ref. 32; i.e., separate epigenetic marks laid down by one epigenetic enzyme may interfere in the localization of a different enzyme for an entirely different epigenetic mark (33)). With this in mind, we chose to continue our epigenetic-based studies using HDACis for the potential double-positive effect on gene transcription by both directly interfering with the removal of histone acetylation posttranslational marks and interfering with DNA CpG methylation (a negative regulator of transcription; ref. 34).

**Figure 2.**
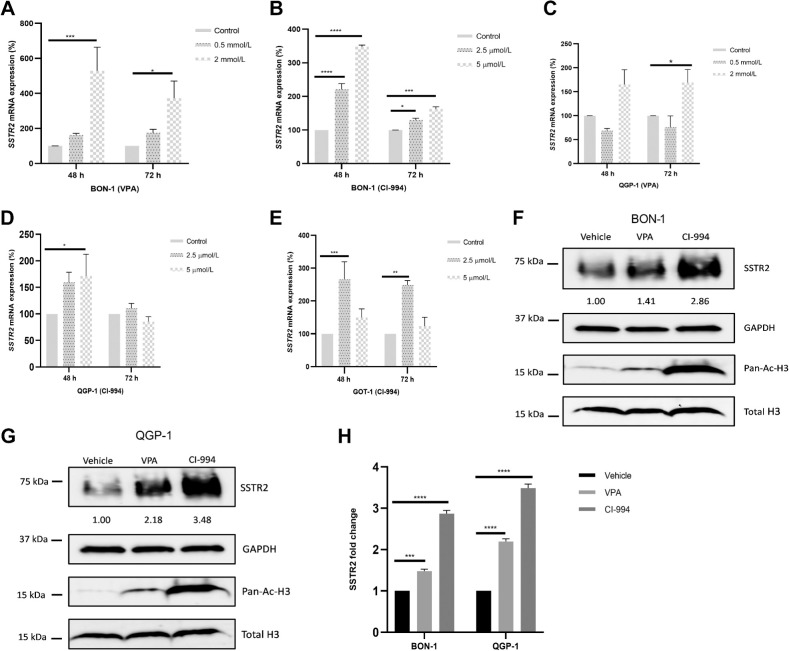
HDACis increase SSTR2 transcription and protein expression in three NET cell lines. **A,** BON-1 cells treated with VPA at 2 mmol/L for 48 hours (*P* = 0.0001) and 72 hours (*P* = 0.0144) significantly increased SSTR2 mRNA expression. **B,** BON-1 cells treated with CI-994 at 2.5 μmol/L for 48 hours (*P* = 0.0001) and 72 hours (*P* = 0.03), and at 5 μmol/L for 48 hours (*P* = 0.0001) and 72 hours (*P* = 0.0001) had a significant increase in SSTR2 expression. **C,** QGP-1 cells treated with VPA at 2 mmol/L for 72 hours had a significant increase in SSTR2 expression (*P* = 0.0379). **D,** QGP-1 cells treated with CI-994 at 5 μmol/L for 48 hours (*P* = 0.0392) had a significant increase in SSTR2 expression. **E,** GOT-1 cells treated with CI-994 at 2.5 μmol/L for 48 hours (*P* = 0.0008) and 72 hours (*P* = 0.0022) showed a significant increase in SSTR2 expression. **F,** HDACis VPA and CI-994 increased SSTR2 protein expression in BON-1 cells 1.4- and 2.8-fold, respectively, with a concomitant increase in H3-histone acetylation at 48 hours. **G,** HDACis VPA and CI-994 increased SSTR2 protein expression in QGP-1 cells 2.18- and 3.48-fold, respectively, with a concomitant increase in histone acetylation at 48 hours. **H,** Graphical representation of the FC in total SSTR2 protein expression in BON-1 and QGP-1 cells. Data are expressed as mean ± SEM, (*n* = 3). *, *P* < 0.05; **, *P* < 0.01; ***, *P* < 0.001; ****, *P* < 0.0001.

We found upregulation of *SSTR2* gene and protein expression levels after treatment with the epidrugs VPA and CI-994 in all three cell lines in a dose- and time-dependent manner. Protein expression studies were performed using an SSTR2 antibody that was validated by SSTR2 knockdown experiments (Supplementary Fig. S1A and S1B). In BON-1 cells, both VPA and CI-994 treatment at 48 and 72 hours yielded a significant, dose-dependent increase in SSTR2 expression (Fig. 2A and B). In QGP-1 cells, VPA treatment yielded a significant increase in SSTR2 expression at 72 hours (Fig. 2C) and CI-994 treatment at 48 hours compared with the controls (Fig. 2D). In GOT-1 cells, lower treatment concentrations of CI-994 significantly increased SSTR2 expression at both 48 and at 72 hours (Fig. 2E). We found a significant 1.4- and 2.8-fold increase in total SSTR2 protein expression with VPA and CI-994, respectively, with a concomitant increase in histone acetylation in the BON-1 cell line at 48 hours, confirming the target and mode of action of the drugs (Fig. 2F). Similarly, in the QGP-1 cell line, a 2.2- and 3.5-fold increase was seen with both HDACis (Fig. 2G). Graphical representation of the fold changes (FC) in the BON-1 and QGP-1 cell lines are shown in Fig. 2H.

### HDACi treatment increases SSTR2 surface expression in three NET cell lines

**Figure 3.**
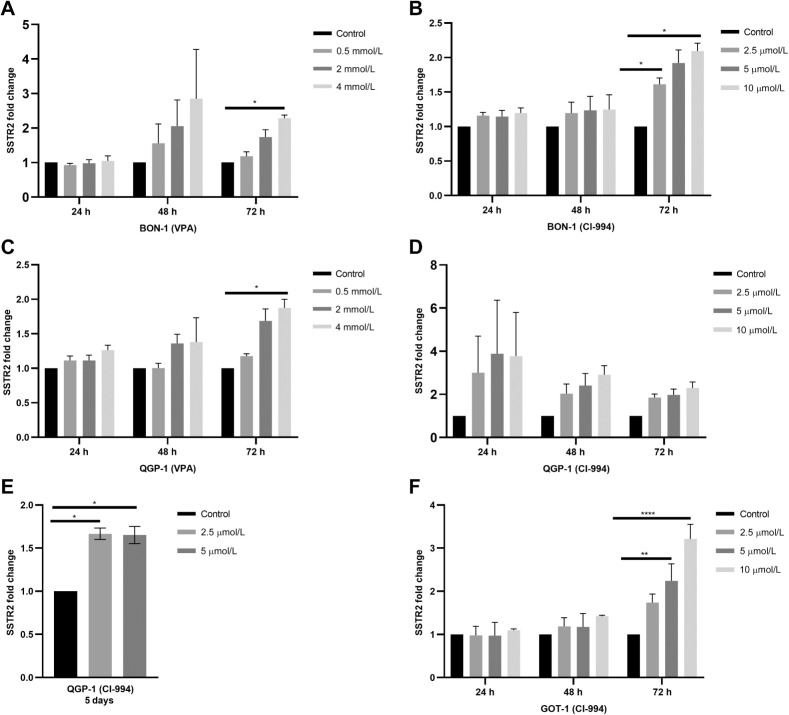
HDACis upregulate SSTR2 surface expression in three NET cell lines. **A** and **B,** SSTR2 surface expression in BON-1 cells was determined by flow cytometry analysis. Cells treated with both VPA at 4 mmol/L (*P* = 0.0103) and CI-994 at 2.5 μmol/L (*P* = 0.0416) and 10 μmol/L (*P* = 0.0205) for 72 hours showed a significant increase in SSTR2 expression. **C,** QGP-1 cells treated with VPA at 4 mmol/L for 72 hours (*P* = 0.0366) had a significant increase in SSTR2 expression. **D,** Cells treated with CI-994 had no significant increase in SSTR2 expression. **E,** QGP-1 cells treated with CI-994 at 2.5 μmol/L (*P* = 0.0159) and 5 μmol/L (*P* = 0.0174) for 5 days showed a significant increase in SSTR2 expression. **F,** GOT-1 cells treated with CI-994 at 5 μmol/L (*P* = 0.0051) and 10 μmol/L (*P* < 0.0001) for 72 hours had a significant increase in SSTR2 expression. Data are expressed as mean ± SEM, (*n* = 3). *, *P* < 0.05; **, *P* < 0.01; ***, *P* < 0.001; ****, *P* < 0.0001.

At 72 hours, using flow cytometry analysis, we found a significant dose- and time-dependent increase in SSTR2 surface expression in BON-1 cells with both VPA and CI-994 treatment (Fig. 3A and B), and in QGP-1 cells treated with VPA (Fig. 3C). The increase in cell surface expression in QGP-1 with CI-994 was not significant (Fig. 3D); however, when investigating the long-term effects of a 5-day treatment, we found a significant increase in SSTR2 surface expression in CI-994–treated QGP-1 cells (Fig. 3E). At 72 hours, we found a significant increase in SSTR2 surface expression in CI-994–treated GOT-1 cells (Fig. 3F). In addition, using receptor saturation assay, we report significantly increased MESF, i.e., SSTR2 per cell, after treatment with CI-994 in both BON1 and QGP1 cells (Supplementary Fig. S1C and S1D).

### HDACi treatment increases SSTR2 surface protein expression in a xenograft model expressing low basal SSTR2 levels

**Figure 4.**
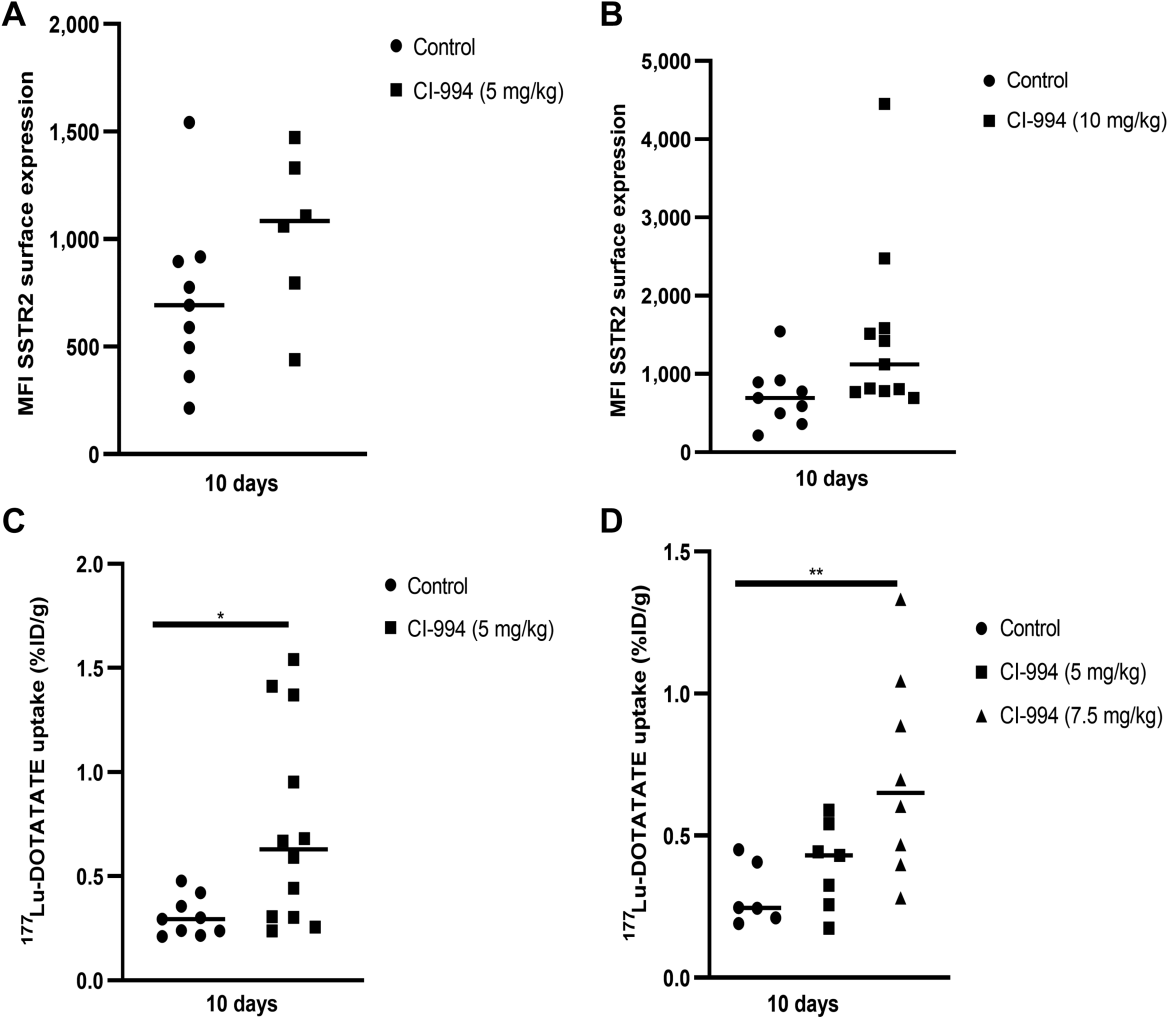
HDACis increase SSTR2 surface expression and ^177^Lu-DOTATATE uptake in QGP-1 xenograft model. **A,** There was an increase in SSTR2 expression with CI-994 (5 mg/kg) after 10 days of treatment, though the change was not significant (control *n* = 9, treated *n* = 6). **B,** There was an increase in SSTR2 expression with CI-994 (10 mg/kg) after 10 days of treatment, though the change was not significant (control *n* = 9, treated *n* = 11). **C,** Higher ^177^Lu-DOTATATE uptake in QGP-1 tumors indicates increased SSTR2 expression and increased therapeutic potential. A significant increase of ^177^Lu-DOTATATE was observed in tumors of mice treated with CI-994 for 10 days at 5 mg/kg (*P* = 0.0175, control *n* = 9, treated *n* = 12). **D,** Persistent uptake of ^177^Lu-DOTATATE after a 72-hour interval without CI-994 was found, with a significant increase observed in mice treated with CI-994 at 7.5 mg/kg for 10 days (*P* = 0.0092, control *n* = 6, CI-994; 5 mg/kg) *n* = 7, CI-994 (7.5 mg/kg) *n* = 8. Each circle, square, or triangle represents one tumor. Data are expressed as mean ± SEM; *, *P* < 0.05; **, *P* < 0.01.

Using a flank xenograft model, we initially performed pilot studies in both BON-1 and QGP-1 tumors using CI-994 treatment for 3, 6, or 10 days, to determine the best SSTR2 surface response, depending on duration of drug exposure. We found a trend towards higher SSTR2 surface expression in QGP-1 tumors treated with CI-994 treatment for 10 days at both 5 and 10 mg/kg (Fig. 4A and B). We demonstrated low basal SSTR2 expression in QGP-1 control tumors compared with BON-1 control tumors (Supplementary Fig. S2A and S2B), and therefore chose to continue our subsequent experiments using the QGP-1 xenograft model as a representative model of human low- to negative-SSTR2–expressing NETs. Similar to our FACS results, we showed increased SSTR2 expression by IF staining after CI-994 treatment in QGP-1 xenograft tumors compared with the controls (Supplementary Figs. S2B–S3D). IHC staining of QGP-1 tumors was positive for chromogranin A (Supplementary Fig. S2D) with a high Ki67 proliferation index (Supplementary Fig. S2E). These results confirm that QGP-1 is a representative model for high-grade, low-SSTR2–expressing NET. We observed a significant decrease in the % of Ki67 with CI-994 treatment (Supplementary Figs. S2I, S3A, and S3B).

Importantly, we did not find any significant changes in SSTR2 surface upregulation with another HDACi, VPA, at any timepoint in our *in vivo* studies (3, 6, or 10 days of treatment). Therefore, we did not use this drug for further preclinical investigation (Supplementary Fig. S2F, shown at 10 days of treatment). Body weight changes shown in Supplementary Fig. S2G and S2H correspond to *in vivo* studies shown in Fig. 4A and B. These demonstrated minimal toxicity of CI-994 treatment in mice undergoing 10 days of daily intraperitoneal treatment at 5 mg and 10 mg/kg, when compared with the control group.

### CI-994 treatment increases ^177^Lu-DOTATATE uptake within QGP-1 xenograft tumors

To assess whether *in vitro* increases in both SSTR2 expression and plasma membrane quantity translate to increased uptake *in vivo*, we treated QGP-1–engrafted mice with CI-994 and quantified ^177^Lu-DOTATATE uptake in tumors. We found a significant increase in ^177^Lu-DOTATATE uptake in tumors treated with CI-994 for 10 days (Fig. 4C). Our findings confirm increased cell-surface SSTR2 after HDAC inhibition that resulted in increased accumulation of ^177^Lu-DOTATATE in QGP-1 tumors. Similarly, we found a significant increase in ^177^Lu-DOTATATE uptake in tumors pretreated with CI-994 (7.5 mg/kg) after a-72-hour interval without additional CI-994 treatment. This indicates the potential to persistently upregulate SSTR2 expression, even after removal of the drug, facilitating tumor uptake of ^177^Lu-DOTATATE (Fig. 4D). However, this ^177^Lu-DOTATATE uptake was not found to be significant at the lower dose of CI-994 (5 mg/kg). The tumor-to-organ ratio of ^177^Lu-DOTATATE uptake showed at least a 2-fold increase in CI-994 (5 mg/kg)-treated QGP-1 tumors compared with control tumors, which represents a key feature for imaging contrast in SSTR2-targeted PRRT (Supplementary Fig. S4A and S4D). Minimal ^177^Lu-DOTATATE uptake was seen in other organs indicating minimal toxicity to these organs; the higher uptake in kidneys is due to the elimination of Lu-DOTATATE through the urinary tract (Supplementary Fig. S5A). Body weight changes in the corresponding *in vivo* studies (Fig. 4C and D), which demonstrated limited HDACi toxicity, are shown in Supplementary Fig. S4B and S4E. The individual changes in tumor volume in the above studies are shown in Supplementary Fig. S4C and S4F, showing identical tumor sizes at the start of HDACi treatment, with no significant differences between treatment and control groups. This indicates a minimal effect of HDACi alone on tumor growth within the 10 days of intraperitoneal injections.

### CI-994 pretreatment combined with ^177^Lu-DOTATATE promotes tumor regression in an SSTR2-deficient xenograft model compared with standard therapy

**Figure 5.**
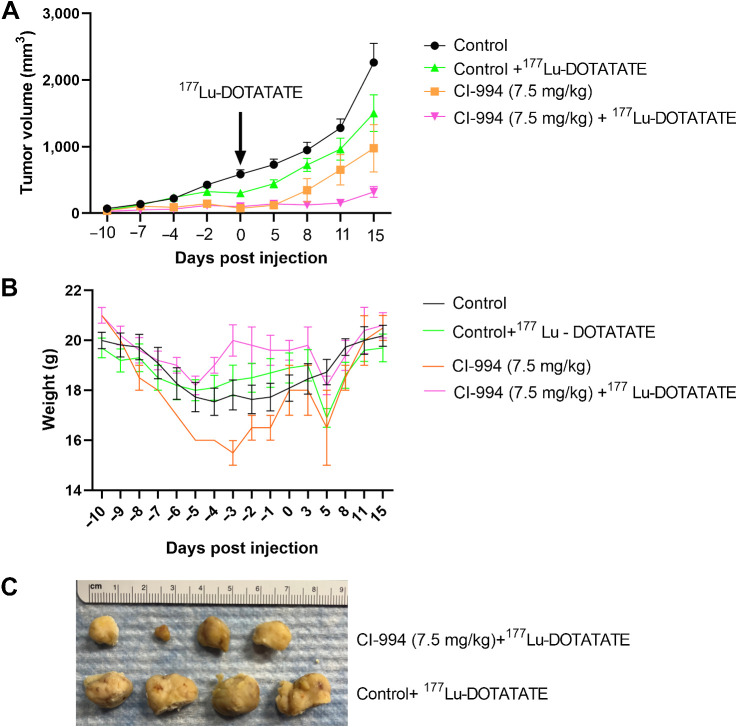
Increased ^177^Lu-DOTATATE uptake induced by CI-994 treatment promotes tumor regression in QGP-1 xenograft model. **A,** Tumor volume (mm^3^) ± SEM from start to the end of pretreatment with CI-994 and injection of 30 MBq ^177^Lu-DOTATATE at day 0 (arrow). Mice were then observed for tumor growth responses to ^177^Lu-DOTATATE for 15 days. Day 11: control vs. CI-994 + ^177^Lu-DOTATATE (*P* = <0.0001); control + ^177^Lu-DOTATATE vs. CI-994 + ^177^Lu-DOTATATE (*P* = 0.0008). Day 15: control versus CI-994 + ^177^Lu-DOTATATE (*P* <0.0001); control + ^177^Lu-DOTATATE versus CI-994 + ^177^Lu-DOTATATE (*P* = 0.0028). **B,** Weight (g) ± SEM of mice from start to the end of treatment with CI-994 and ^177^Lu-DOTATATE. Control *n* = 18, Control +177Lu-DOTATATE *n* = 18, CI-994 *n* = 4, CI-994 + 177Lu-DOTATATE *n* = 10. Data are expressed as mean ± SEM. **C,** Representative images of QGP-1 xenograft tumors collected after euthanasia, comparing the combination CI-994 +^177^Lu-DOTATATE therapy group to the control + ^177^Lu-DOTATATE group.

Using an identical 10-day CI-994 pretreatment model, the mice that received a single intravenous administration of 30 MBq of ^177^Lu-DOTATATE after CI-994 pretreatment demonstrated a significant reduction in tumor growth compared with the control group (*P* < 0.0001) and to the group receiving standard therapy, that is, 30 MBq ^177^Lu-DOTATATE alone (*P* = 0.0028; Fig. 5A). This was confirmed 11 and 15 days after ^177^Lu-DOTATATE injections. And, the effects of combination therapy were additive, that is, there was no interaction effect between CI-994 and ^177^Lu-DOTATATE. The clear difference in tumor growth after 5 days of ^177^Lu-DOTATATE therapy between the two CI-994–pretreated groups signaled a strong response to ^177^Lu-DOTATATE. Tumor growth was not slowed in the ^177^Lu-DOTATATE–only treatment group, potentially due to SSTR2 deficiency. Notably, the combined CI-994 and ^177^Lu-DOTATATE treatment appeared well-tolerated, with limited toxicity as evidenced by some changes in mouse body weight as observed in other studies (35), with no visible toxicity signs at the time of euthanasia (Fig. 5B). Tumors of mice treated with combined CI-994 and ^177^Lu-DOTATATE were significantly smaller compared with tumors of mice treated ^177^Lu-DOTATATE alone (Fig. 5C). Furthermore, Pan H3 staining revealed increased open chromatin (red foci, Pan H3) in tumors treated with CI-994 in comparison to control tumors. And increased DNA damage (green foci, γH2AX) was observed in these CI-994–pretreated tumors compared with control after ^177^Lu-DOTATATE therapy (Supplementary Fig. S6A–S6D). This increase in DNA damage was found to be significant (*P* < 0.001; Supplementary Fig. S5B).

## Discussion

Our study results show that pretreatment with HDACis increase SSTR2 surface expression *in vitro* and *in vivo*, resulting in increased ^177^Lu-DOTATATE tumor uptake. This translates into improved therapeutic efficacy of PRRT in a SSTR2-deficient PNET model.

Clinically, patients with high-grade, SSTR2-negative PNETs are refractory to PRRT, with poor responses to chemotherapy (7) and, most importantly, have no other therapeutic options. Because patients with low-grade, SSTR2-positive metastatic NETs have shown significant progression-free survival benefits in response to SSTR2-targeted PRRT (9), we proposed to investigate *in vivo* whether by increasing SSTR2 surface levels we can improve tumor responsiveness to PRRT for clinical translation in patients with high-grade, receptor-negative metastatic PNETs.

In our study, we demonstrate for the first time the clinical relevance of using ^177^Lu-DOTATATE as a targeted therapy in a receptor-deficient *in vivo* PNET model. Our ultimate goal was to discover and characterize novel, SSTR2-directed, epidrug-based treatments to improve responses to PRRT. Changes in epigenetic marks have been demonstrated in various cancers, including PNETs (36, 37), and because these changes are reversible, they serve as targets for the modulation of gene expression. We found that higher NET grades correlated with increased *SSTR2* promoter methylation levels in an NIH NET patient cohort, which provides an explanation for the silenced expression of SSTR2, as shown in other datasets (38, 39). In high-grade PNETs, SSTR2 expression seems lost due to epigenetic gene-silencing mechanisms (14, 40, 41, 42); and studies have reported a reversal of these mechanisms by treating NET cell lines with DNMTis and HDACis (43), thus inducing DNA hypomethylation and histone acetylation, respectively (37, 44, 45). In our *in vitro* studies, we observed that QGP-1, BON-1, and GOT-1 cell lines have different baseline expression levels of SSTR2 (QGP-1 < BON-1 < GOT-1), consistent with prior reports (26). To the best of our knowledge, we are the first to show that higher DNA methylation correlates with lower SSTR2 expression in these three NET cell lines. We elicited a particularly strong response to HDACi in QGP-1, which harbors hypermethylated *SSTR2* promoter levels and low SSTR2 expression. Accordingly, we judged QGP-1 to be most representative of the high-grade, SSTR2-negative human PNETs and used it for our *in vivo* therapeutic studies. We demonstrated a significant and strong dose-dependent upregulation in SSTR2 cell surface expression at 72 hours and at 5 days of HDACi treatment, but not at shorter timepoints. These results indicate that both time course and dose play crucial roles in regulating SSTR2 transcription using epidrug-based treatments. Others have reported similar results for upregulation of SSTR2 mRNA in BON-1 and GOT-1 with HDACi treatments (12, 14, 42), as well as with demethylating agents (39). In an *in vitro* study in BON-1 and NCI-H727 cells (14), the reversibility of *SSTR2* gene expression upregulation after HDACi (VPA and CI-994) withdrawal returned to baseline within 24 hours. *In vivo*, our results confirm that effects induced by low doses of HDACi treatment (5 mg/kg) for a short duration were largely and rapidly reversible. Interestingly, the higher dose of CI-994 (7.5 mg/kg) led to persistent upregulation of SSTR2 expression, even after withdrawal of the drug for 3 days, showing a significant increase in ^177^Lu-DOTATATE uptake within tumors. Similar results were found by *Evans* and colleagues (39), demonstrating increased SSTR2-targeted imaging tracer uptake in BON-1 xenografts after treatment with the demethylating agent guadecitabine.

To the best of our knowledge, our study is the first to report *in vivo* therapeutic results with combined epidrug- ^177^Lu-DOTATATE in a PNET model harboring low basal SSTR2 expression. Our results demonstrate significant upregulation of SSTR2 surface expression and ^177^Lu-DOTATATE uptake after HDACi treatment in QGP-1 tumor xenografts. Most relevant was the significant tumor regression seen in HDACi-pretreated mice in response to the ^177^Lu-DOTATATE therapy, when compared with both control and ^177^Lu-DOTATATE-only treated mice. Importantly, this combined, sequential therapeutic effect of HDACi CI-994 and ^177^Lu-DOTATATE therapy was additive. Furthermore, the ^177^Lu-DOTATATE-only treatment group had a poorer response than the CI-994–only treatment group. We hypothesize that this may be due to the low basal SSTR2 expression in our QGP-1 xenograft model, which would explain the poor response to treatment with ^177^Lu-DOTATATE alone. This *in vivo* model is thus representative of the poor response to PRRT in humans with high-grade, receptor-negative NETs. In addition, we observed extensive DNA damage with the uptake of ^177^Lu-DOTATATE into tumors, with the higher absorbed dose translating into increased DNA damage and leading to better outcomes (combined treatment group); as shown by using γH2AX, a marker for DNA damage induced by the biological effect of ^177^Lu-DOTATATE PRRT (46).

The major strength of our study is its translational relevance, as the therapeutic efficacy of PRRT has only been proven for models characterized by high baseline SSTR2 surface expression levels. Our results suggest that receptor-deficient tumors, such as in our QGP-1 model, may respond to PRRT using ^177^Lu-DOTATATE when pretreated with selective epidrugs. These epidrugs will increase SSTR2 expression, thus expanding the bounds of PRRT clinical efficacy for use in treating patients with high-grade SSTR2-negative PNETs. Furthermore, in line with our *in vivo* studies, these epidrug treatments have been demonstrated to be safe in patients, as demonstrated in a clinical imaging study analyzing the safety of the HDACi vorinostat (47). However, more studies are necessary to understand the toxicity profiles in patients undergoing combined epidrug –^177^Lu-DOTATATE therapy.

Our study has a few limitations. Unfortunately, to date, there are no representative high-grade metastatic NET models for GEP-NETs, whether based on human cell lines or orthotopic human tumor tissues. We utilized three NET cell lines for our *in vitro* studies to best represent all possible responses to our investigational drugs due to varying degrees of SSTR2 surface expression. To overcome this limitation in our *in vivo* studies, we selected the QGP-1 model, shown to harbor the lowest SSTR2 surface expression, so as to best represent human high-grade, receptor-negative NET behavior. For future studies, we are currently formulating human-derived 3D organoid and xenograft models. This will potentially also allow us to overcome the limitation of short *in vivo* evaluation of therapeutic efficacy, with the possibility for additional ^177^Lu-DOTATATE dosing.

In conclusion, our preclinical data demonstrate that pretreatment with the HDACi CI-994 improves ^177^Lu-DOTATATE therapy, compared with PRRT alone in models of SSTR2-deficient NETs. Our study forms the basis for a clinical trial testing the therapeutic efficacy of HDACi CI-994 pretreatment in combination with ^177^Lu-DOTATATE therapy in patients with high-grade, SSTR2-negative metastatic PNETs.

## Supplementary Material

Figure S1, Figure S2, Figure S3, Figure S4, Figure S5, Figure S6, Table S1 Supplementary Figures and Tables with Legend
